# Serum electrolytes in children with heart failure on diuretic therapy at Jakaya Kikwete Cardiac Institute, Dar es Salaam, Tanzania: a cross-sectional study

**DOI:** 10.11604/pamj.2024.48.181.41906

**Published:** 2024-08-16

**Authors:** Samuel Raymond Mushi, Zawadi Edward Kalezi, Alphonce Nsabi Simbila

**Affiliations:** 1 Muhimbili University of Health and Allied Sciences, Dar es Salaam, Tanzania,; 2 Department of Pediatric Cardiology, Jakaya Kikwete Cardiac Institute, Dar es Salaam, Tanzania,; 3 Department of Emergency Medicine, Muhimbili University of Health and Allied Sciences, Dar es Salaam, Tanzania

**Keywords:** Serum electrolytes, heart disease, children, Tanzania

## Abstract

**Introduction:**

diuretic therapy is among the cornerstones of the management of heart failure in children with acquired or congenital heart diseases (CHD). Electrolyte derangements have been reported by various studies to be among the most common side effects following diuretic therapy. Despite that, there is limited documentation on the magnitude of this problem in our setting. This study, therefore, aimed to identify clinical characteristics and determine the magnitude of electrolyte abnormalities among children with heart failure who were on diuretic therapy at Jakaya Kikwete Cardiac Institute, Tanzania.

**Methods:**

this hospital-based cross-sectional study was conducted among children with heart failure admitted at Jakaya Kikwete Cardiac Institute (JKCI), Dar es Salaam, Tanzania. A pretested structured questionnaire was used to collect data on socio-demographics, clinical characteristics, and levels of serum electrolytes.

**Results:**

a total of 385 children whose ages ranged from 1 month to 18 years were enrolled in this study. Most of them (271 (70%)) were below 5 years and their median age was 2.1 (IQR=0.8-6) years. Ventricular septal defect (VSD) was the most common acyanotic CHD, observed in 122 children out of 385 (31.6%). Nearly half (186 (48%)) of the children were in class IV of the Ross Heart Failure Classification. Slightly more than half of the study participants (202 (53%)) and 182 (47%) of the children had hyponatremia and hypocalcemia respectively. Only 8% (32/385) had hypokalemia. There was no observed association between hyponatremia and furosemide dosage (P=0.33) in children in this study.

**Conclusion:**

a significant number of children with heart failure on diuretic therapy had hyponatremia and hypocalcemia hence, routine electrolytes monitoring is recommended.

## Introduction

Heart failure is common among children with heart conditions. It complicates such congenital or acquired conditions as rheumatic heart disease and cardiomyopathies [1]. Its medical management includes the use of diuretics, angiotensin-converting enzymes (ACE) inhibitors, and vasoactive agents, with or without inotropic support [2]. Diuresis and improved perfusion are immediate goals of care in children with heart failure [1]. Loop diuretics are the first-line therapy for diuresis in the treatment of heart failure in children [3,4]. They exert their effect by inhibiting sodium, potassium, and chloride cotransport (NKCC) in the proximal part of the loop of Henle. The proximal tubule resorbs 15 to 25% of the filtered sodium load [5]. Furosemide is commonly used in pediatrics. Its immediate hemodynamic effect is modulated by the secretion of renin from the juxta glomerular apparatus [5]. It has a direct kaliuretic effect in the loop of Henle causing potassium loss. Moreover, the addition of thiazide-like diuretics improves diuresis at the cost of increased risk of serum electrolyte abnormalities [3]. The occurrence of electrolyte abnormalities as adverse effects is not unusual following diuretic therapy [1,3,5]. Hyponatremia and hypokalemia are the most observed abnormalities [6]. Magnesium and calcium could unnoticedly be affected too. They play a vital role in bone formation in children [5,6]. Their extreme derangements can cause neurological complications including seizures [6].

Along with furosemide, spironolactone is commonly used in pediatrics [7]. Despite being a “potassium-sparing” diuretic, in combination with other diuretics it may still cause hypokalemia. Contrarily, in children receiving ACE inhibitors potassium levels should be closely monitored due to the risk of causing hyperkalemia [8]. A study in the USA reported hyperkalemia among patients in early diuretic therapy inclusive of spironolactone [7]. However, throughout treatment, hypokalemia became more common. In Nigeria, one study observed that combinational diuretic therapy with spironolactone was able to maintain electrolyte balance [9]. Chronic diuretic therapy causes hypertrophy of the distal nephrons thus increasing sodium reabsorption leading to diuretic resistance [5,10]. This effect can be counteracted by dose increments or combining diuretics. It also accelerates symptoms of heart failure inducing syndrome of inappropriate ADH secretion (SIADH) with free water retention [10]. At the JKCI little is known about electrolyte derangements and clinical characteristics of children with heart failure who are treated with diuretics. This study, therefore, aimed to determine the magnitude of electrolyte abnormalities and document the clinical characteristics of children with heart failure who were on diuretic therapy.

## Methods

**Study design and setting:** this was a hospital-based cross-sectional study conducted in a pediatric cardiology unit at the Jakaya Kikwete Cardiac Institute (JKCI), Tanzania from January 2023 through June 2023.

**Study population:** all children aged between 1 month and 18 years diagnosed with either congenital or acquired heart disease admitted to the pediatric cardiology unit of the JKCI during the study period were enrolled in the study. Children with diarrhea, vomiting, acute kidney injury, and those who denied consent to participate in the study were excluded.

**Sample size determination:** the sample size was calculated using Kish and Leslie formula, and the estimated minimum sample size was 385. Data collection was done using a standardized structured questionnaire for each study participant.

**Study variables:** the socio-demographic and clinical data collected included age, sex, date of admission, address, anthropometric measurements (weight, length/ height), clinical chemistry (electrolytes; sodium, potassium, magnesium, and calcium), serum creatinine, blood urea nitrogen, diagnoses, and details of the treatment regimen.

**Sample collection and testing:** a blood sample of approximately 2 milliliters (mls) was aseptically drawn from the antecubital fossa of each admitted child for clinical chemistry as the standard of care.

**Data management and analysis:** data entry and cleaning were done using SPSS software version 25. Means, medians, frequencies, and proportions of each variable were determined. Contingency tables were then constructed for bivariate analysis. The probability p-value <0.05 was considered to be statistically significant.

**Ethical approval:** to conduct the study was obtained from the Ethics Review Committee of the Jakaya Kikwete Cardiac Institute (Ref.No.AB.123/307/01J/2).

## Results

There were 387 children with heart diseases admitted to the pediatric cardiology unit of the JKCI during the study period. A total of 385 children whose data were included in the final analysis met the inclusion criteria and were enrolled into the study. Out of 385 children recruited, 70% (271/385) were below 5 years and their median age was 2.1 (IQR=0.8-6) years, with half of them 198 (51%) being males. One-third 122 (32%) of the children had ventricular septal defect (VSD) followed by atrioventricular septal defect (AVSD) in 65 (17%) and patent ductus arteriosus (PDA) in 39 (10%). Nearly half 186 (48%) of the study participants had class IV heart failure (Table 1). Half (202/385) of the studied children had hyponatremia while only 8% (32/385) and 4% (15/385) had hypokalemia and hypomagnesemia respectively. Forty-seven percent (182/385) of the children had hypocalcemia (Table 2). However, on bivariate analysis, there was no association between the electrolyte derangements and the dosage of furosemide (Table 3).

**Table 1:** socio-demographic and clinical characteristics of children with congenital or acquired heart diseases who are on diuretic therapy at the Jakaya Kikwete Cardiac Institute

Variable	Category	Frequency (%)
Age (year)	≤ 1	129 (33)
	>1-5	142 (37)
	>5	114 (30)
	**Median (IQR)**	**2.1 (0.8-6)**
Weight (kg)	Mean ± SD	13.1 ± 11.6
Sex	Male	198 (51)
	Female	187 (49)
Caretaker education level	Primary	96 (25)
	Secondary	209 (54)
	Higher level	80 (21)
Diagnosis	Atrial septal defect (VSD)	31 (8)
	Ventricular septal defect (VSD)	122 (32)
	Atrioventricular septal defect (AVSD)	65 (17)
	Patent ductus arteriosus (PDA)	39 (10)
	Coarctation of aorta (CoA)	2 (0.5)
	Tetralogy of fallot (TOF)	45 (12)
	Truncus arteriosus (TA)	17 (4)
	Tricuspid atresia (TA)	2 (0.5)
	Rheumatic heart disease (RHD)	55 (14)
	Cardiomyopathies	3 (1)
	Infective endocarditis (IE)	4 (1)
Heart failure classification	Class II	112 (29)
	Class III	87 (23)
	Class IV	186 (48)

SD: Standard deviation

**Table 2:** serum electrolytes levels of children with congenital or acquired heart diseases who are on diuretic therapy at the Jakaya Kikwete Cardiac Institute

Variable	Category	Frequency (%)
Sodium level	Severe/ Moderate hyponatremia	64 (17)
	Mild hyponatremia	138 (36)
	Normal level	183 (47)
Potassium level	Hypokalemia	32 (8)
	Normal level	311 (81)
	Hyperkalemia	42 (11)
Calcium level	Hypocalcemia	182 (47)
	Normal level	200 (52)
	Hypercalcemia	3 (1)
Magnesium level	Hypomagnesemia	15 (4)
	Normal level	290 (75)
	Hypermagnesemia	80 (21)

**Table 3:** association between electrolyte abnormalities and diuretics dosage among children with congenital or acquired heart diseases who are on diuretic therapy at the Jakaya Kikwete Cardiac Institute

Variable	Category	Furosemide dosage		P-value (Chi^2^)
		<1-2mg/kg/day	>2mg/kg/day	
**Sodium level**	Severe/Moderate hyponatremia	23 (14)	41 (18)	0.33
	Mild hyponatremia	64 (40)	74 (33)	
	Normal level	74 (46)	109 (49)	
**Potassium level**	Hypokalemia	13 (8)	19 (9)	0.48
	Normal level	134 (83)	177 (79)	
	Hyperkalemia	14 (9)	28 (12)	
**Calcium level**	Hypocalcemia	74 (46)	108 (48)	0.86
	Normal level	86 (53)	114 (51)	
	Hypercalcemia	1 (1)	2 (1)	
**Magnesium**	Hypomagnesemia	8 (5)	7 (3)	0.63
	Normal level	121 (75)	169 (76)	
	Hyperkalemia	32 (20)	48 (21)	

## Discussion

This study identified clinical characteristics and determined the magnitude of electrolyte abnormalities among children with heart failure who were on diuretic therapy at Jakaya Kikwete Cardiac Institute, Tanzania. In a period of six months 385 children were treated for heart failure with diuretics. More than half of them were males, were under five years, had different types of congenital heart diseases and were in class IV of the Ross Heart failure classification. Hyponatremia and hypocalcemia were observed in approximately half of the study participants; however, furosemide dosage had no association with the observed results. A few of them had hypokalemia and hypomagnesemia. Most of the studied children were under five years of age and had congenital cardiac malformations with ventricular septal defect being the leading underlying diagnosis. Unlike adult heart failure, heart failure in children is mostly caused by structural congenital heart diseases followed by cardiomyopathies. Similar observations have been reported by studies from other parts of the world [11]. The presence of heart failure necessitated treatment with, among other drugs, diuretics while waiting for definitive corrective interventions such as cardiac surgery. A large proportion of children in this study had hyponatremia although most of them were on furosemide whose common side effect is hypokalemia. This is higher than the proportions reported by other studies in Nigeria [6,9]. It may be explained by the fact that furosemide causes electrolyte derangements by various mechanisms. When using furosemide hyponatremia may occur as an adverse effect of inhibiting sodium, potassium, and chloride cotransport (NKCC) in the proximal part of the loop of Henle hence blocking resorption of a significant amount of filtered sodium inducing diuresis.

In addition, hyponatremia is a biomarker of poor short- and long-term outcomes in heart failure patients. More than half of the children in this study were very ill, and in class IV of Ross Heart Failure Classification. Hyponatremia may have been a product of various pathophysiological neurohormonal and inflammatory mechanisms other than diuretic therapy. Nevertheless, loop diuretics and fluid restriction remain the mainstay treatments of dilutional hyponatremia in children with heart failure [12]. Only eight percent of study participants were found to have hypokalemia which is a frequently reported electrolyte abnormality in loop diuretic therapy [5,7,9]. This may have been due to the use of a regimen that included a combination of potassium-sparing diuretics as a standard of care at our institute. Similar results were reported in Nigeria by Sadoh and his colleagues [9]. Although a small number of children had hypomagnesemia, for successful treatment of hypokalemia, the magnesium deficit must be reversed. Concomitant hypomagnesemia aggravates hypokalemia and renders it refractory to treatment with potassium.

If left untreated both hypokalemia and hypomagnesemia may trigger cardiac arrhythmias which could worsen heart failure. We also observed hypocalcemia in nearly half of our study participants which could possibly be due to induced changes in electrochemical gradient by diuretic drugs between interstitial cells and renal tubules hence reducing the calcium absorption. The above results are comparable to what has been reported by Tabansi and his colleagues [6]. Calcium is essential for cardiac contraction and relaxation. The presence of hypocalcemia has been proven to influence cardiac function resulting in poor cardiac contractility. Unnoticed and uncorrected hypocalcemia in children with heart failure who are on diuretic therapy could result in myocardial contractile dysfunction and worsen their cardiac condition. In some rare cases hypocalcemic cardiomyopathy has been documented as a manifestation or a complication of heart failure in patients with low serum calcium levels. In such instances calcium supplementation is required for normalization of left ventricular function and better treatment outcome [13,14]. This study was conducted at a single center which might have affected its generalizability. However, the Jakaya Kikwete Cardiac Institute serves as the national referral center for paediatric cardiac conditions and receives enough patients from all over Tanzania and nearby countries in the region. Therefore, the patients included were likely to provide a true representation of the intended population of children with heart conditions.

## Conclusion

The observed magnitude of electrolyte abnormalities was high among children with heart failure who were on diuretic therapy in our setting. Hyponatremia and hypocalcemia were the leading abnormalities followed by hypokalemia and hypomagnesemia respectively. Despite being the most used diuretic agent expected to cause hypokalemia as its side effect, furosemide had no association with the electrolyte abnormalities observed in the studied heart failure children. Regular serum electrolytes measurement other than potassium in children with heart failure who are on diuretic therapy is therefore important.

### What is known about this topic

- Diuretic therapy is associated with electrolyte imbalance;
- Diuretic therapy is among the cornerstones in the management of heart failure in children.

### What this study adds

- This study highlights the magnitude and types of electrolyte abnormalities among heart failure children who are on diuretic therapy at the Jakaya Kikwete Cardiac Institute;
- This study provides valuable baseline data to guide further research on electrolyte abnormalities in children with heart failure who are on diuretic therapy, using alternative study designs.
